# Association mapping of quantitative resistance to charcoal root rot in mulberry germplasm

**DOI:** 10.1371/journal.pone.0200099

**Published:** 2018-07-06

**Authors:** Marian Vincent Pinto, Poornima H. S., Rukmangada M. S., Triveni R., V. Girish Naik

**Affiliations:** Molecular Biology Laboratory– 1, Central Sericultural Research and Training Institute, Mysuru, Karnataka, India; Huazhong University of Science and Technology, CHINA

## Abstract

Outbreaks of root rot disease in the productive South Indian sericulture belt have threatened the sustainability of the industry. *Macrophomina phaseolina* (Tassi) Goid. causing charcoal rot is the predominant pathogen to which all productive mulberry cultivars are susceptible. The present study was undertaken to identify molecular markers associated with charcoal rot resistance in mulberry. A mapping panel comprising 214 diverse entries from the Indian germplasm collection was assessed for charcoal rot resistance by artificial inoculation. Resistance to the pathogen was observed in 20 entries, and 51 were found to be moderately resistant. A total of 773 alleles generated across 105 SSR loci and 20,384 AFLP markers generated using 32 *Eco*RI-NN and *Mse*I-CNN primer combinations were used in genetic analysis. The panel was weakly structured with two subpopulations. However, most entries were found to be admixtures. Survival of cuttings and number of roots per sapling were associated with root rot resistance. Association mapping was performed using different linear mixed models. Five AFLP markers explaining 9.6–12.7% of the total phenotypic variance were found to be significantly (*p* < 0.05) associated with root rot resistance. Significant associations were also detected in four AFLP markers for survival of cuttings, and these markers explained 10.7–14.2% of the total phenotypic variance. These markers should be validated using mapping populations derived from contrasting biparental combinations by linkage analysis for use in marker-assisted gene pyramiding for durable resistance. The resistant genotypes identified in this study will substantially contribute to genetic improvement of mulberry for charcoal rot resistance and can be integrated into conventional breeding programmes.

## Introduction

Mulberry (*Morus* L. spp.) is indispensable in the sericulture industry, as the domesticated silkworm *Bombyx mori* (Linnaeus, 1758) feeds exclusively on the foliage of these plants to meet its nutritional requirements. Mulberry cultivation accounts for approximately 40% of the total cost of cocoon production (S1 Table). Hence, it is obvious that mulberry improvement for higher productivity, leaf quality, adaptability to different agroclimatic conditions, abiotic stress tolerance and disease resistance is imperative to sustainably enhance the quality and yield of silk. Mulberry is a perennial and is grown as a lowbush, highbush or small tree plantation for silkworm rearing. A well-maintained garden can give good yields for 15–20 years [1]. Root diseases are a major problem in mulberry cultivation, as managing them is quite a challenge when compared with foliar diseases. Perennial nature of the crop, resilience of soilborne pathogens, and their persistence in soil as spores and sclerotia provide a congenial atmosphere for the establishment of infectious agents and inoculum build-up [2, 3]. Of late, mulberry root rot outbreaks have become a serious threat in four South Indian states–Karnataka, Andhra Pradesh, Tamil Nadu and Telangana [4]. These four states cumulatively account for approximately 80% of the mulberry raw silk production in the country [5].

Various types of root rots, such as dry rot caused by *Fusarium solani* (Mart.) Sacc. and *F*. *oxysporum* Schlecht., black rot caused by *Botryodiplodia theobromae* Pat. [= *Lasiodiplodia theobromae* (Pat.) Griff. and Maubl.] and charcoal rot caused by *Macrophomina phaseolina* (Tassi) Goid. [= *Rhizoctonia bataticola* (Taubenh.) E.J.Butler], have been reported in mulberry from India [6–8]. Occurrence of disease complexes due to infection by more than one root rot-causing pathogen and association of root-knot nematode [*Meloidogyne incognita* (Kofoid and White, 1919) Chitwood, 1949] with root rot have also been reported [9, 10]. However, it was found that *M*. *phaseolina* is the most prevalent pathogen in the South Indian sericulture belt [4, 9]. Most mulberry cultivars are prone to charcoal rot disease and can cause up to 35% leaf yield loss, reduction in leaf size, deterioration of leaf quality, and plant mortality [11]. These in turn adversely affect profitability in sericulture [12].

Many chemical and biological methods have been recommended for the control of mulberry root rots [13]. The use of chemicals is undesirable due to residual toxicity on silkworms [14]. Indiscriminate use of fungicides comes at a cost to the environment and human health [15]. Furthermore, it leads pathogens to evolve resistance [16]. Biological approaches for the control of soilborne diseases are not very successful due to various factors, such as variability in performance and poor efficacy under optimal conditions for disease development, stemming from the complex and dynamic host plant × pathogen × biocontrol agent × environment interactions [17]. Non-availability of root rot-resistant mulberry cultivars has made the use of chemicals an unavoidable necessity. As such, genetic improvement by breeding for resistance is the only tenable option. Genetic resistance is the most effective, cost-efficient and environmentally friendly method for disease control [18]. A good example for this is breeding for leaf rust resistance in spring bread wheat undertaken by CIMMYT, Mexico, which has resulted in limiting crop losses to an insignificant level over the decades [19]. The benefit-cost ratio for these efforts was estimated to be 27:1 [20].

Plant breeders have mostly relied on germplasm resources and crop wild relatives for useful genetic variants to create novel gene combinations in crop improvement programmes [21]. India holds a rich germplasm collection of 1291 mulberry accessions of both indigenous and exotic origin [22]. Significant variability in agronomically important traits is observed in the germplasm [23–26]. To date, in India, only 20 mulberry cultivars have been screened for their disease response to *M*. *phaseolina*, and all were found to be susceptible [11]. However, Hongthongdaeng [27] reported that the mulberry cultivar Pai and F_1_ hybrids Pai × Noi No. 6, 18, 33 and 36 exhibited resistance to root rot disease in Thailand. This indicates the availability of root rot-resistant sources in the germplasm. Therefore, screening a representative subset of mulberry germplasm is important to identify charcoal rot-resistant accessions.

Mulberry breeding is constrained by its long juvenile period, outcrossing nature, heterozygosity, *etc*. [28]. The development of a new mulberry cultivar requires approximately 15 years on average [28]. Modern breeding approaches such as marker-assisted selection (MAS) are more efficient and precise for targeted trait improvement and can be undertaken once the quantitative trait loci (QTLs) for the trait of interest have been mapped and validated [29, 30]. MAS has been successfully applied in breeding bacterial blight resistance in rice [31], *Fusarium* head blight resistance in wheat [32], potato late blight resistance [33], *etc*. Association mapping allows simultaneous screening of germplasm and mining QTLs. The method takes advantage of historical recombination events in a population that bring about decay in linkage disequilibrium (LD) to detect causative variants in tight linkage with the trait of interest [34]. Employing diverse germplasm resources for mining QTLs ensures that the complete genetic variability underlying the trait in the gene pool is accounted for [34]. Sampling germplasm panels to retain maximum genetic diversity in a minimum size has a normalizing effect that reduces population structure and LD between unlinked loci [35]. Therefore, such panels are ideal for association analysis. Krishnan [36] selected a ‘panel of diverse germplasm’ (PDG) comprising 300 entries using the SimEli sampling strategy. This PDG represents the entire genetic diversity available in the Indian mulberry germplasm collection. The present study was undertaken to evaluate the PDG against *M*. *phaseolina* and to identify molecular markers associated with charcoal root rot resistance.

## Materials and methods

### Plant materials

Cuttings of the PDG entries were obtained from the *ex situ* gene bank at Central Sericultural Germplasm Resources Centre, Hosur, and the nursery was raised according to recommended practices [37].

The PDG was established as a small tree (90 cm crown height) plantation under augmented randomized complete block design [38] at Central Sericultural Research and Training Institute (CSRTI), Mysuru (12° 15′ 38.6″ N, 76° 37′ 30.6″ E). The plot with red sandy loam soil was divided into 5 blocks, each comprising 3 sub-blocks. Every entry was represented by 4 ramets in a sub-block, with a spacing of 1.5 m between plants and 1.8 m between sub-blocks. Victory-1 (indigenous) and Kousen (exotic) were grown as check cultivars. An end guard row of Victory-1 was planted to eliminate border effects. Poor rooting entries were propagated by grafting buds onto Victory-1 stem stocks [39] and established in the plot. The plantation was maintained as per recommended practices [37] but with a biannual pruning schedule.

### Phenotyping for root rot resistance

#### Preparation of inoculum

The most virulent isolate of *M*. *phaseolina* from mulberry–MP-5 [4] was used in the study. Sorghum grains were soaked for 18 h in water. The water was drained, and 100 g of the soaked grain was weighed into 300 ml culture bottles. These bottles were autoclaved at 121°C for 1 h. Ten discs (8 mm diameter) cut from 4-day-old cultures of the pathogen grown on potato dextrose agar (PDA) were used to inoculate the sterilized sorghum. The bottles were incubated at room temperature for 30 days with intermittent mixing. The sorghum was completely colonized by the pathogen and darkened with microsclerotia at the end of the incubation period and was used for inoculation of mulberry saplings.

#### Evaluation of disease response

Four-month-old saplings were carefully uprooted from nursery beds and were planted in earthenware pots (18 cm diameter) with 2 kg of sterilized red sandy loam soil thoroughly mixed with 1 bottle of the fungal inoculum in July 2015 (see [40] for metrological data). The pots were arranged in three randomized complete blocks under open-field conditions. Every accession was represented by one inoculated pot and one uninoculated pot (control) in a block. Pots were irrigated with 300 ml of water once every two days during the dry period. The total number of leaves per plant, number of wilted leaves per plant, number of dead plants per accession, weight of the whole root system per plant (g) and weight of the healthy portion of the root system per plant (g) were recorded 90 days after inoculation, and disease indices (leaf wilting, healthy root, root rot and plant mortality percentages) were calculated [41]. Based on the percentage of root rot, the disease response was categorized on a scale of 0–5 (Table 1). A set of 10 randomly selected accessions was phenotyped for a second time during February–May 2016 (see [42] for metrological data). Analysis of variance (ANOVA) and other statistical computations were performed using R 3.4.0 [43].

**Table 1:** Categorization of disease responses in the mulberry germplasm.

Scale	Root Rot %	Disease Response
0	< 1%	Highly resistant
1	1–< 26%	Resistant
2	26–< 51%	Moderately resistant
3	51–< 76%	Moderately susceptible
4	76–< 90%	Susceptible
5	> 90%	Highly susceptible

#### Pathogen recovery and molecular identification

Among the charcoal rot infected accessions, 6 were randomly selected, and roots from 1 sapling per accession were thoroughly washed with tap water. Root bits 5 mm in length were surface sterilized by dipping in 2% sodium hypochlorite solution for 2 min. The root bits were then thoroughly rinsed in sterilized deionised water to remove the bleach and inoculated onto PDA plates supplemented with streptomycin sulphate (100 mg/l). The plates were incubated at room temperature for 2–5 days. Hyphal tips emerging from the root bits were transferred to fresh PDA plates and were incubated for 5–7 days to obtain axenic cultures. DNA was extracted from mycelia of the stock culture and reisolated pathogen using a commercial kit (HiPurA Fungal DNA Purification Kit: HiMedia Laboratories Pvt. Ltd., Mumbai, India) according to the manufacturer’s instructions. RAPD fingerprinting was performed using 8 informative arbitrary primers (OPA-03, OPD-13, OPD-18, OPG-17, OPM-04, OPQ-20, OPR-15, OPR-18: Operon Technologies Inc., Alameda, CA, USA) as described by Naik and Dandin [44] to ascertain the identity of the reisolated pathogen. The reproducibility of RAPD fingerprinting was assessed by performing replicate PCRs.

#### Testing of ‘tails’

The ‘tails’ (5 each of resistant and highly susceptible genotypes) identified after phenotyping the panel for disease response to *M*. *phaseolina* were assessed for their reaction to other root rot pathogens using the virulent isolates *F*. *solani* ‘FS-13’, *F*. *oxysporum* ‘FO-20’ and *B*. *theobromae* ‘BT-2’ [4]. Inoculum preparation and phenotyping were performed as described previously, from August–November 2016 (see [42] for metrological data).

### Phenotypic data on yield and propagation parameters

Data on mulberry germplasm characterization for leaf yield per plant (kg) and propagation parameters such as survival of cuttings (%), number of roots per sapling, fresh root weight per sapling (g), dry root weight per sapling (g), longest root length per sapling (cm) and root volume per sapling (ml) were compiled from ‘Catalogue on mulberry (*Morus* spp.) germplasm’ (Vol. 1–5) [23–26].

### DNA extraction

Genomic DNA was extracted from fresh young mulberry leaves collected from the PDG plot using a commercial kit (HiPurA Plant Genomic DNA Miniprep Purification Kit: HiMedia Laboratories Pvt. Ltd., Mumbai, India) according to the manufacturer’s instructions. An aliquot (2 μl) of each DNA sample was electrophoresed on 1% agarose gel in 1× TAE at 4 V/cm for 1 h [45]. The DNA was quantified using a microvolume spectrophotometer (NanoDrop 2000C: Thermo Fisher Scientific Inc., Wilmington, DE, USA). DNA samples that appeared as a single, sharp high-molecular-weight band on the agarose gel, with an A_260_/A_280_ ratio of 1.8–2, were used in genotyping.

### AFLP genotyping

Restriction digestion, adaptor ligation and preselective amplification reactions were performed as per the protocol of Vos *et al*. [46] with minor modifications. Genomic DNA (200–250 ng) was incubated with 5 U each of *Eco*RI-HF and *Mse*I in 40 μl of 1× CutSmart Buffer for 1 h at 37°C. A 10 μl solution containing 5 pmol of *Eco*RI adaptor, 50 pmol of *Mse*I adaptor, 5 mM ATP and 80 cohesive end units of T4 DNA Ligase in 1× CutSmart Buffer was added to the digestion reactions, and incubation was continued for 3 h at 37°C. The restriction–ligation products were diluted two-fold with T_10_E_0.1_ (pH 8.0) buffer.

The preselective amplifications were carried out in 20 μl reaction volumes containing 1× Standard *Taq* Reaction Buffer, 200 μM of each dNTP, 0.3 μM *Eco*RI primer, 0.3 μM *Mse*I-C primer, 0.5 U of *Taq* DNA Polymerase and 2 μl of the diluted restriction–ligation product. The reactions were performed with the following cycling profile: 20 cycles of 94°C for 30 s, 56°C for 1 min, 72°C for 1 min, and a final extension step at 72°C for 7 min. The PCR products were diluted eight-fold with T_10_E_0.1_ (pH 8.0) buffer.

The selective amplifications were performed as per Clarke [47] with minor modifications in 10 μl reaction volumes containing 1× Standard *Taq* Reaction Buffer (with 1.5 mM MgCl_2_), an additional 3.125 mM MgCl_2_, 250 μM of each dNTP, 0.5 μM 5′-labelled *Eco*RI-NN primer (Applied Biosystems Ltd., Woolston, Cheshire, UK), 0.5 μM *Mse*I-CNN primer, 0.5 U of *Taq* DNA Polymerase and 2 μl of the diluted preselective amplification product. The touchdown PCR cycling was carried out as follows: 13 cycles of 94°C for 30 s, 65°C (reduced by 0.7°C/cycle) for 30 s, 72°C for 1 min, followed by 23 cycles of 94°C for 30 s, 56°C for 30 s, 72°C for 1 min, and a final extension step at 72°C for 7 min.

All enzymes used in AFLP genotyping were procured from New England BioLabs Inc., Ipswich, MA, USA. The oligonucleotides used are listed in S2 Table. All preselective and selective amplification reactions were performed on the GeneAmp PCR System 9700 (Applied Biosystems Inc., Foster City, CA, USA), and the ramping speed was limited to 1°C/s. Capillary electrophoresis and scoring of AFLP profiles were performed as described by Clarke [47].

### SSR genotyping

A total of 7 SSR primers designed for *M*. *boninensis* Koidz. [48] and 154 SSR primers designed for *M*. *indica* L. were used in the study (S3 Table). Screening of primers, PCR optimization and genotyping were performed as described by Pinto *et al*. [49]. The resolving power (Rp) of each primer pair was calculated as per Prevost and Wilkinson [50].

The mulberry germplasm was previously characterized with 74 SSR markers [36, 49], which generated a total of 542 alleles across the loci. These genotypic data were also utilized in the present study. Unless stated otherwise, genetic analysis was performed with the combined AFLP and SSR marker dataset, and all markers with > 10% missing data were excluded from analysis.

### Assessment of genetic diversity and kinship

A dissimilarity matrix based on Dice’s coefficient [51] was computed, and cluster analysis was performed by the neighbour-joining method [52] using the software DARwin 6.0.14 [53]. The dendrogram was drawn using FigTree 1.4.2 (http://tree.bio.ed.ac.uk/software/figtree/).

The codominant SSR marker data were used to compute pairwise kinship coefficients [54] among the accessions with SPAGeDi 1.5 [55]. All negative values in the 214 × 214 kinship matrix were set to zero, as suggested by Yu *et al*. [56].

### Population structure analysis

Bayesian clustering was performed to investigate population stratification in the panel using the software *Structure* 2.3.4 [57–59]. The admixture model with correlated allele frequencies was used. All markers were coded as dominant, and ploidy was set to 1. The run length was set to 110,000 MCMC iterations with the fist 10,000 being discarded as burn-in for subpopulations (*K*) ranging from 1–6. Five runs were performed for each *K* value. The optimal number of subpopulations was determined by the method of Evanno *et al*. [60]. *ΔK* was calculated using the web application Structure Harvester 0.6.94 [61]. For the optimum *K*, replicate runs were aligned with the *FullSeach* algorithm implemented in the program *CLUMPP* 1.1.2 [62]. The *outfile* from *CLUMPP* was used to generate a bar plot of the *Structure* results using the web application Structure Plot 2.0 (http://omicsspeaks.com/strplot2/) [63]. Principal component analysis (PCA) was also performed to assess population stratification in the panel using the R package adegenet 2.0.1 [64]. The first and second principal components (PCs), which explained the maximum variance, were plotted to obtain the graphical output. Analysis of molecular variance (AMOVA) was performed according to Excoffier *et al*. [65] using Arlequin 3.5.2.2 [66] to assess the molecular variance within and between *Structure*-defined subpopulations. The program FAMD 1.31 [67] was used to count private alleles in each of the subpopulations.

### Association mapping

Association mapping for charcoal rot resistance was performed using the accession means of healthy root percentage as the response variable. Marker–trait association (MTA) analysis was also performed for survival of cuttings and number of roots per sapling, as these traits were found to be associated with root rot resistance. Various statistical models described by Yu *et al*. [56], namely, simple, Q, K and Q+K, were used for MTA, with subpopulation membership coefficients as fixed covariates and kinship as random effects. MTA was performed using TASSEL 5.2.37 [68] by the general linear model procedure for simple and Q models, whereas compressed mixed linear model with population parameters previously determined [69] was used for K and Q+K models. The *p*-values were corrected for multiple testing by the Benjamini and Hochberg step-up false discovery rate (FDR) controlling procedure [70] implemented in the R package multtest 2.32.0 [71]. The significance threshold for MTA was set at ≤ 0.05. The model with the lowest mean of the squared differences (MSD) between observed and expected *p*-values was selected as the best [72]. Quantile–quantile (Q–Q) plots of the *p*-values were also generated to assess the adequacy of the models in controlling type I errors.

## Results

### Disease response of the mapping panel

**Fig 1:**
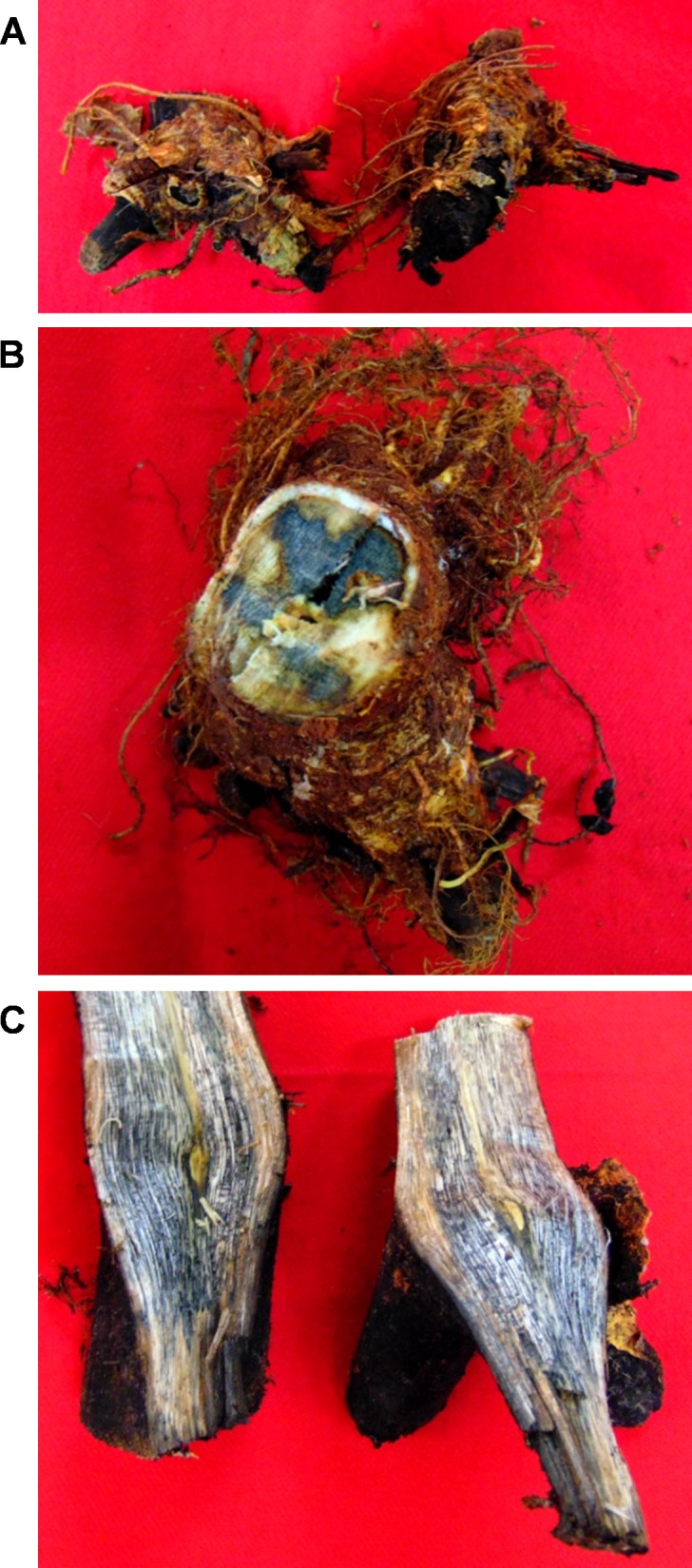
Infected roots–external view (A), traverse section (B) and longitudinal section (C).

A total of 214 entries from the PDG were found to root and could be screened for their reaction to charcoal root rot causing *M*. *phaseolina* by artificial inoculation. These 214 entries constituted the mapping panel. Diverse response to the pathogen was observed (S4 Table; Fig 1). High resistance to the pathogen was not observed, which indicates the absence of qualitative resistance to *M*. *phaseolina* in the set of screened germplasm. However, 20 accessions with less than 26% root rot were classified as resistant. *M*. *cathayana* (Hybrid) had of the fewest infected roots (9.85%), and 2 accessions of *M*. *multicualis* Perr., ME-0168 and ME-0006, had 12.03% and 16.57% root infection, respectively. G-2, a new cultivar recommended for young silkworm rearing, and G-4, an improved cultivar, both bred from *M*. *multicaulis* (♀), were classified as resistant (22.81% root rot) and moderately resistant (35.91% root rot), respectively. Moderate resistance to the infection was observed in 51 accessions; 50 accessions were categorized as moderately susceptible; 19 accessions were classified as susceptible; and 74 accessions were found to be highly susceptible, of which 73 were completely infected by the pathogen. The wild mulberry species *M*. *laevigata* Wall. ex Brandis was represented by 6 accessions that were all found to be highly susceptible. The main effects for disease responses were significant (leaf wilting: *F*_213,426_ = 2.97, *p* = 6.53 × 10^−22^; root rot and healthy root: *F*_213,426_ = 3.64, *p* = 4 × 10^−30^) and negligible between replications (leaf wilting: *F*_2,426_ = 0.23, *p* = 0.8; root rot and healthy root: *F*_2,426_ = 1.42, *p* = 0.24). There were no significant differences between the disease responses of the 10 accessions in the two trials (leaf wilting: *F*_1,18_ = 0.44, *p* = 0.51; root rot and healthy root: *F*_1,18_ = 0.02, *p* = 0.88). Disease responses of the ‘tails’ to other fungal root rot pathogens has been presented in S5 Table. The stock culture and all pathogen reisolates had identical RAPD profiles, thus fulfilling Koch’s postulates [73]. The banding profiles were consistent across PCR replications.

### Phenotypic variability in the mapping panel

**Fig 2:**
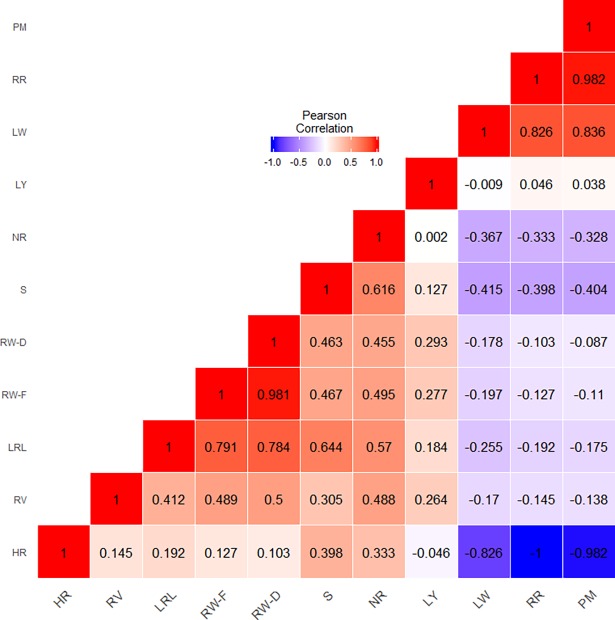
Correlation among various phenotypic traits observed in the mapping panel. RR, root rot; HR, healthy root; LW, leaf wilting; PM, plant mortality; S, survival of cuttings; RW-F, fresh root weight per sapling; RW-D, dry root weight per sapling; NR, number of roots per sapling; RV, root volume; LRL, longest root length per sapling; LY, leaf yield per plant.

**Fig 3:**
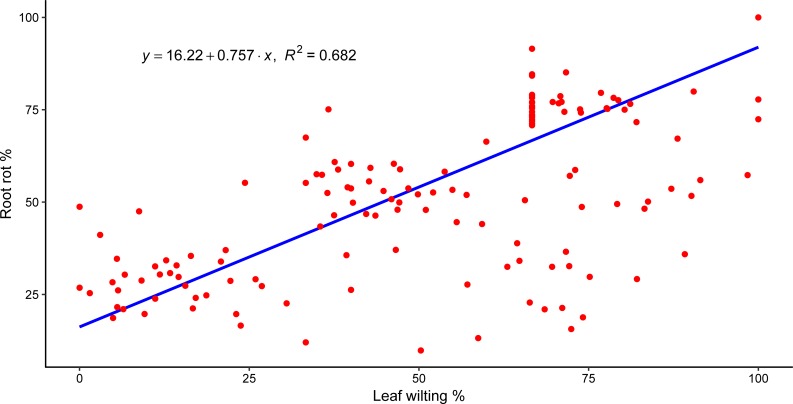
Linear relationship between the leaf wilting and root rot traits.

The summary statistics of the distribution of phenotypic traits in the mapping panel are presented in Table 2. The root rot phenotype had a highly significant (*p* < 0.001) correlation with leaf wilting (*r* = 0.826) and plant mortality (*r* = 0.982). A highly significant (*p* < 0.001) negative correlation was also observed between root rot and survival of cuttings (*r* = –0.398) and the number of roots per sapling (*r* = –0.333) traits (Fig 2). The yield potential of an accession was not correlated with root rot susceptibility. There was a significant linear relationship (*R*^2^ = 0.682; *p* < 0.001) between leaf wilting and root rot percentages (Fig 3).

**Table 2:** Distribution of phenotypic traits in the mapping panel.

Trait	Minimum	Maximum	Mean	SD	Variance	CV%	Skewness	Kurtosis
Root rot (%)	9.85	100	67.24	29.10	846.68	43.27	–0.24	–1.36
Healthy root (%)	0	90.15	32.76	29.10	846.68	88.82	0.24	–1.36
Leaf wilting (%)	0	100	67.39	31.73	1007.04	47.09	–0.57	–0.92
Plant mortality (%)	0	100	53.43	40.29	1623.57	75.42	–0.11	–1.55
Survival of cuttings (%)	4.17	96.67	62.36	22.58	509.64	36.20	–0.89	0.16
Fresh root weight per sapling (g)	0.18	17.87	5.41	4.16	17.30	76.95	0.99	0.05
Dry root weight per sapling (g)	0.08	6.76	1.85	1.54	2.38	83.56	1.06	0.41
Number of roots per sapling	1.56	15.33	7.32	2.69	7.23	36.71	0.33	–0.08
Root volume (ml)	0.13	23.33	4.13	4.12	17	99.73	1.60	3.07
Longest root length per sapling (cm)	10	45.17	24.54	7.39	54.60	30.11	0.29	–0.60
Leaf yield per plant (kg)	0.10	7.08	1.87	1.29	1.66	68.96	1.45	2.67

### DNA profiling

A total of 31 primer pairs of the 161 screened were found to be polymorphic. PCR conditions were optimized for these primers (Table 3) and were used in DNA fingerprinting of the mapping panel. These markers amplified a total of 231 alleles (7.45 alleles/locus) in the size range 49–420 bp. M2SSR23 amplified the maximum of 17 alleles, whereas M2SSR51 and M2SSR88 amplified only 2 alleles each. A total of 87 rare alleles (present in < 5% of the accessions) and 3 common alleles (present in > 98% of the accessions) were observed. The resolving power of the 31 SSR markers ranged from 0.07 to 3.66 and totalled 60.8. AFLP fingerprinting of the mapping panel was performed using 32 selective primer combinations (S2 Table). A total of 20,384 fragments were amplified by these selective primers. Of these, 10,988 (53.9%) had a frequency < 5%, and 202 had a frequency > 98%.

**Table 3:** Optimized PCR conditions and SSR marker polymorphism observed in the mapping panel.

Sl. No.	Marker	P	T_A_	C	V	Size Range (bp)	N_A_	N_R_	N_C_	Rp
1.	M2SSR1	0.5	44.2	30	3	217–257	5	2	0	1.72
2.	M2SSR10	0.25	47.6	30	5	123–159	11	5	0	2.46
3.	M2SSR101	0.25	46.8	30	10	370–420	3	0	1	0.53
4.	M2SSR102	0.5	46.9	30	3	201–213	4	1	0	1.37
5.	M2SSR103	0.25	48.2	30	10	285–355	8	3	0	0.86
6.	M2SSR107	0.25	53.3	30	3	247–262	4	1	0	1.99
7.	M2SSR12	0.5	46.8	30	3	270–278	4	1	0	0.68
8.	M2SSR20	0.5	48.8	30	3	232–298	9	5	0	2.85
9.	M2SSR23	0.25	47.8	30	10	214–286	17	9	0	3.5
10.	M2SSR36	0.25	47.2	30	3	196–208	6	3	0	1
11.	M2SSR41	1	46.9	30	10	250–286	6	1	0	2.13
12.	M2SSR51	0.5	47.1	32	10	209–227	2	1	1	0.07
13.	M2SSR54A	0.25	48.1	35	5	215–277	7	3	0	1.06
14.	M2SSR64B	0.5	46.4	30	5	170–217	11	6	0	1.25
15.	M2SSR68	0.5	46.1	30	5	192–216	7	1	0	2.7
16.	M2SSR72	0.5	47.6	30	5	198–218	5	2	0	1.81
17.	M2SSR81	0.25	47.1	35	3	247–262	4	1	0	2.16
18.	M2SSR82	0.5	44.9	30	3	186–216	7	2	0	2.55
19.	M2SSR87	0.25	49.7	30	5	225–263	10	3	0	3.07
20.	M2SSR88	0.5	48.2	30	5	275–293	2	0	1	0.28
21.	M2SSR89A	1	46.5	30	3	200–238	8	2	0	1.85
22.	M2SSR9	0.6	49.9	32	10	315–383	5	0	0	1.84
23.	M2SSR93	0.5	44.9	30	10	235–247	5	0	0	1.85
24.	MulSSR22S	0.25	56.8	35	10	258–297	8	2	0	3.33
25.	MulSSR59	0.25	59.8	25	5	151–181	8	2	0	2.73
26.	MulSSR85	0.5	56.8	30	5	291–357	13	4	0	3.66
27.	Mos0031	0.5	52	30	5	49–109	9	6	0	1.32
28.	Mos0157-1	0.5	51	30	10	104–146	6	5	0	0.49
29.	Mos0157-2	0.25	49	25	3	242–284	10	1	0	3.51
30.	Mos0288	0.25	47	35	5	118–160	13	8	0	2.9
31.	Mos0340-2	0.25	50	30	5	94–146	14	7	0	3.26

P, optimized concentration of primers (pmol); T_A_, optimized annealing temperature (°C); C, optimized number of PCR cycles; V, volume of PCR products loaded onto the gels (μl); N_A_, number of alleles; N_R_, number of rare alleles; N_C_, number of common alleles; Rp, resolving power.

### Genetic diversity, kinship and population stratification

**Fig 4:**
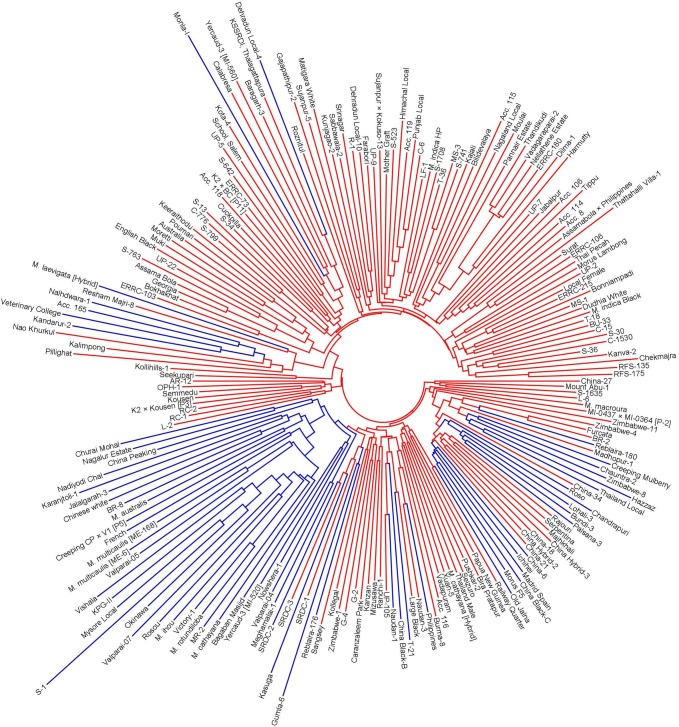
NJ dendrogram of the mapping panel. The *Structure*-defined subpopulation Q1 is coloured red, and Q2 is coloured blue.

Dice’s dissimilarity coefficients among the mulberry accessions ranged from 0.125 to 0.751. The average dissimilarity was found to be 0.446. Georgia and Assama Bola were closely related, and the pair S-1 (*M*. *alba* L.) and Nao Khurkul (*M*. *laevigata*) was the most divergent. The panel was clustered into 2 broad groups (G1 and G2) by the NJ algorithm, comprising 114 and 100 entries (Fig 4).

Ritland’s kinship coefficients calculated based on the SSR marker data ranged from 0 to 0.9318 among the accessions. In the 214 × 214 kinship matrix, 28,058 (61.27%) pairs had a value of 0, 12,188 (26.61%) pairs had a value ≤ 0.05, and 5,386 (11.76%) pairs had a kinship coefficient in the range > 0.05 to ≤ 0.3. Only 164 (0.36%) pairs were highly related to each other, with kinship coefficients in the range > 0.3 to < 0.95.

**Fig 5:**
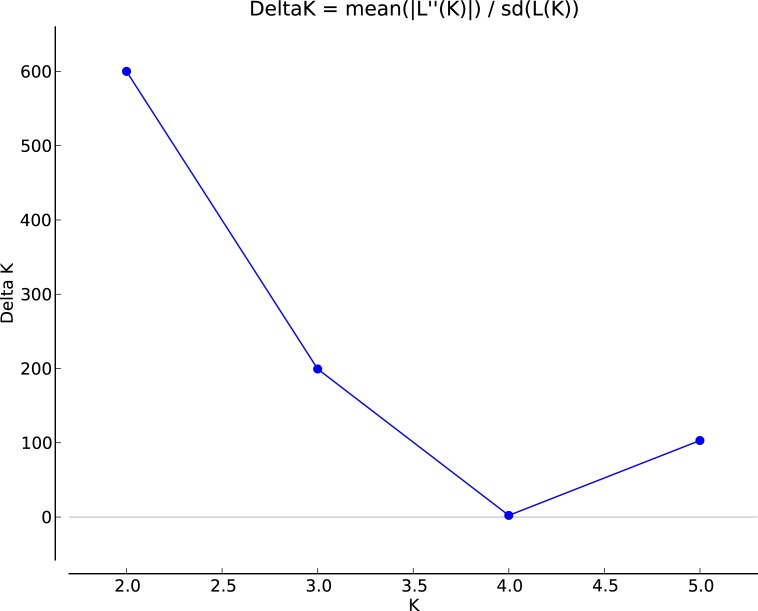
Variation in *ΔK* values across different subpopulation numbers.

**Fig 6:**
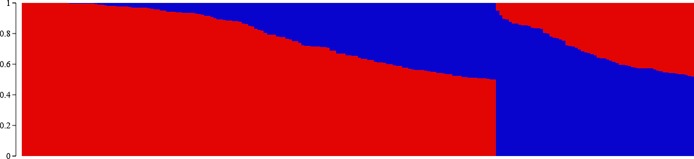
Proportion of subpopulation membership of the 214 diverse mulberry accessions inferred by *Structure*.

**Fig 7:**
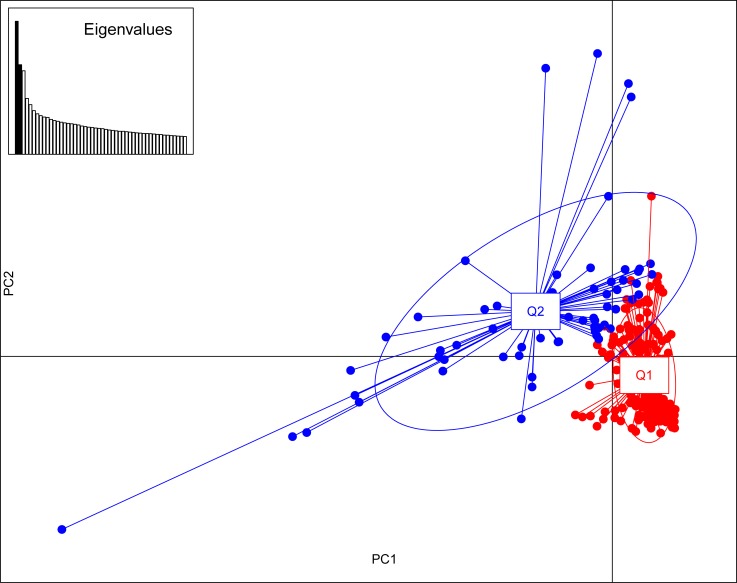
Population stratification in the mapping panel deciphered by PCA. **Inset: Eigenvalues of the principal components.** The *Structure*-defined subpopulation Q1 is coloured red, and Q2 is coloured blue.

Population stratification in the mapping panel was best captured by *Structure* at *K* = 2 (Fig 5). The first subpopulation (Q1) comprised 151 accessions, and 63 accessions were clustered in the second subpopulation (Q2). The exotic cultivar Kousen and 60 indigenous accessions had a membership coefficient > 0.9 in Q1, of which 25 accessions had a membership coefficient ≥ 0.99. In Q2, 2 exotic accessions had a membership coefficient > 0.9. Other than this, most of the accessions were admixtures (Fig 6). As many as 59 accessions had a membership coefficient in the range of 0.4–0.6. The first two PCs that captured 7.17% of the total molecular variance in the mapping panel could delimit the entries into 2 groups with some overlaps (Fig 7), which is in agreement with the *Structure* result. NJ clustering also corroborated the *Structure* and PCA results. The NJ cluster G1 had 107 entries representing Q1 and 7 entries from Q2. The G2 cluster had 100 entries, of which 56 were from Q2. AMOVA revealed that 92.55% molecular variance was contained within *Structure*-defined subpopulations, and 7.45% was partitioned between the subpopulations. The fixation index Φ_ST_ was calculated to be 0.07453, which indicates a weak population structure. A total of 1623 and 2670 alleles were private within Q1 and Q2, respectively.

### Marker–trait association analysis

**Fig 8:**
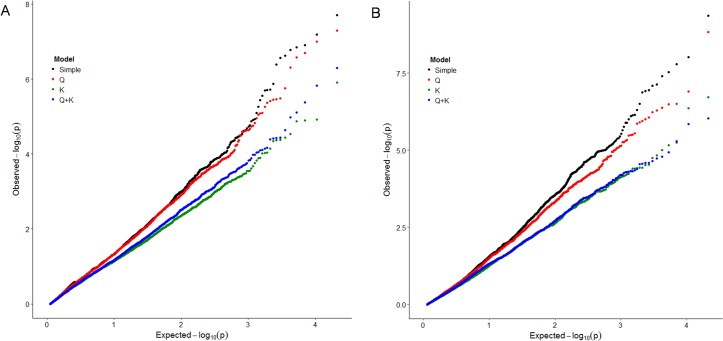
Distribution of expected and observed *p*-values of different statistical models used in marker–trait association analysis of charcoal rot resistance (A) and survival of cuttings (B).

Of the 4 statistical models tested, the simple model performed poorly. Correction for population stratification with the Q matrix from *Structure* did not appreciably reduce the number of false associations. However, the model accounting for familial relatedness with the K matrix from SPAGeDi and a combined Q+K model worked equally well for controlling FDR, as judged by the MSD values (Table 4) and Q-Q plots (Fig 8). A total of 5 AFLP markers were found to be significantly associated with charcoal rot resistance. These markers could explain 9.6–12.7% of the total phenotypic variation in the trait (*R*^2^) and had an allele frequency of 0.132–0.401. The K model could identify only 1 marker associated with charcoal rot resistance, which was also identified by the Q+K model. Four AFLP markers (*R*^2^ = 10.7–14.2%) with allele frequencies in the range 0.052–0.728 were significantly associated with survival of cuttings. The markers E-TG/M-CAG–116 and E-AA/M-CTC–224 were found to be significantly associated with the survival of cuttings in both the K and Q+K models (Table 5). No significant MTAs for the number of roots per sapling trait were found.

**Table 4:** Marker–trait associations and mean of the squared differences between expected and observed *p*-values for different statistical models used in association mapping.

Model	Charcoal Rot Resistance		Survival of Cuttings		Number of Roots per Sapling	
	MTA	MSD	MTA	MSD	MTA	MSD
Simple	78	0.005884	362	0.005116	20	0.007020
Q	34	0.005592	240	0.004971	0	0.006992
K	1	0.002223	4	0.002713	0	0.003795
Q+K	5	0.002368	2	0.002999	0	0.004103

MTA, marker–trait associations; MSD, mean of the squared differences between expected and observed *p*-values.

**Table 5:** Marker loci significantly associated with charcoal rot resistance and survival of cuttings.

Trait	Model	Marker	*p*-value	Adjusted *p*-value	*R*^2^	Allele Frequency
Charcoal rot resistance	K	E-AA/M-CAG–317	1.23 × 10^−6^	0.026	0.119	0.401
	Q+K	E-TG/M-CAG–30	5.14 × 10^−7^	0.011	0.127	0.198
		E-AC/M-CAA–496	1.52 × 10^−6^	0.016	0.115	0.132
		E-AA/M-CAG–317	4.18 × 10^−6^	0.029	0.106	0.401
		E-AA/M-CTG–31	7.77 × 10^−6^	0.041	0.098	0.164
		E-AA/M-CAA–234	1.04 × 10^−5^	0.044	0.096	0.392
Survival of cuttings	K	E-TG/M-CAG–116	4.42 × 10^−7^	0.005	0.142	0.217
		E-AA/M-CTA–342	5.64 × 10^−6^	0.036	0.114	0.052
		E-TG/M-CAA–179	6.86 × 10^−6^	0.036	0.111	0.123
		E-AA/M-CTC–224	9.87 × 10^−6^	0.042	0.107	0.728
	Q+K	E-TG/M-CAG–116	1.42 × 10^−6^	0.015	0.124	0.217
		E-AA/M-CTC–224	5.20 × 10^−6^	0.037	0.109	0.728

## Discussion

### Population structure and interrelationships in the mapping panel

The heterozygous and outcrossing nature of mulberry translates into a high level of phenotypic variation within the segregating progeny. Therefore, mulberry is mainly propagated clonally to ensure uniformity, which is advantageous for cultural operations on a commercial scale. Rooting ability is one of the main criteria for the selection of an improved mulberry cultivar in tropical sericulture. Most tropical cultivars have a good rooting ability and can be conveniently multiplied using stem cuttings. Temperate cultivars, polyploids and wild species, which generally do not root well, are propagated by grafting [28]. Furthermore, it has also been reported that establishing *M*. *serrata* Roxb. in non-native eco-climatic conditions is difficult [74]. The PDG comprises 300 entries from 17 countries and represents 10 *Morus* spp. Of these, 96 entries were exotic, 26 were classified as *M*. *laevigata*, and *M*. *serrata* was represented by 7 entries. A total of 214 entries (S4 Table) were found to root and could be utilized in the present study. Krishnan [36] reported that genetic distance among the PDG entries was in the range 0.071–0.865, with an average of 0.554. Based on the results of the present study, it can be concluded that not much genetic diversity has been lost, even though 86 entries of the PDG were not a part of the mapping panel. Moreover, screening poor rooting accessions for root rot resistance is irrelevant because they are propagated by grafting onto stocks of cultivars with good rooting ability [28], and therefore lack true to type root system.

Germplasm collections consist of genetic resources sampled from different populations and are therefore invariably structured to various extents [75, 76]. Bayesian clustering by *Structure* could identify two subpopulations in the mapping panel. NJ clustering and PCA also support this result. Q1 and Q2 contained 7.69% and 12.66% private alleles, respectively. AMOVA showed that the variance between the subpopulation was very low (7.45%). The fixation index (Φ_ST_ = 0.07453) also indicates a weak population structure. Except for 60 indigenous accessions having a higher membership coefficient in Q1, geographic and taxonomic affinities were not reflected in the Bayesian clustering. From these results and as expected, it is very clear that the two subpopulations are admixed to a great extent. All mulberry accessions in the mapping panel, other than 6 entries representing the wild species *M*. *laevigata*, belong to cultivated species. The cultivated species have dispersed from their centre of origin and established in different countries, including India, and were also introduced from secondary sources at different points in time or bred by crossing naturalized/adapted varieties with introduced materials. No reproductive barrier exists among *Morus* spp. They freely intercross, even among species with different ploidy levels [77, 78], and hence, we cannot expect population stratification. The weak population structure makes this set of germplasm ideal for association mapping.

The AM panel also comprised the ‘kernel’, which consists of accessions frequently used in breeding programmes in India over the past five decades and improved cultivars. Mutants and full-sib, half-sib and first-cousin relationships exist in these accessions [79], and the same is reflected in the kinship matrix based on Ritland’s coefficient.

### Phenotyping for root rot resistance

Having a reliable and efficient technique for screening disease reactions is quite important for the assessment of resistance. Although sick plot screening is considered to be a more realistic method for the evaluation of disease response [80], it may not be very reliable due to non-uniform concentration and non-random distribution of inoculum within and between fields [81]. Variability in the virulence of pathogenic isolates between fields also affects the disease manifestation [82]. Moreover, the method may not be suitable for screening a perennial such as mulberry. Toothpick inoculation is another technique commonly used in annuals, wherein inoculum is introduced through the stalks [83]. This method cannot be implemented in mulberry because it is woody in nature. Furthermore, the method is unsatisfactory because it does not simulate the natural infection process, and the level of disease development is usually less when compared to infection through roots [83]. The results obtained in the present study indicate that artificial inoculation of mulberry saplings in pots was quite reliable in terms of reproducibility and logistically feasible for large-scale screening of germplasm. This is the first study in mulberry wherein a large set of germplasm was assessed for disease response to *M*. *phaseolina*, resulting in the identification of 20 accessions with genetic resistance to charcoal rot. These accessions can be integrated into conventional breeding programmes and used as donor parents for genetic improvement of elite cultivars for charcoal rot resistance.

### Traits associated with root rot resistance

A highly significant positive correlation was observed between root rot and leaf wilting (*r* = 0.826; *p* < 0.001) in the present study (Fig 2, Fig 3). Therefore, leaf wilting can be used as a predictor variable to assess disease progression in terms of root rot percentage in the field. Such estimates will be important for taking up timely control measures on the required scale.

**Fig 9:**
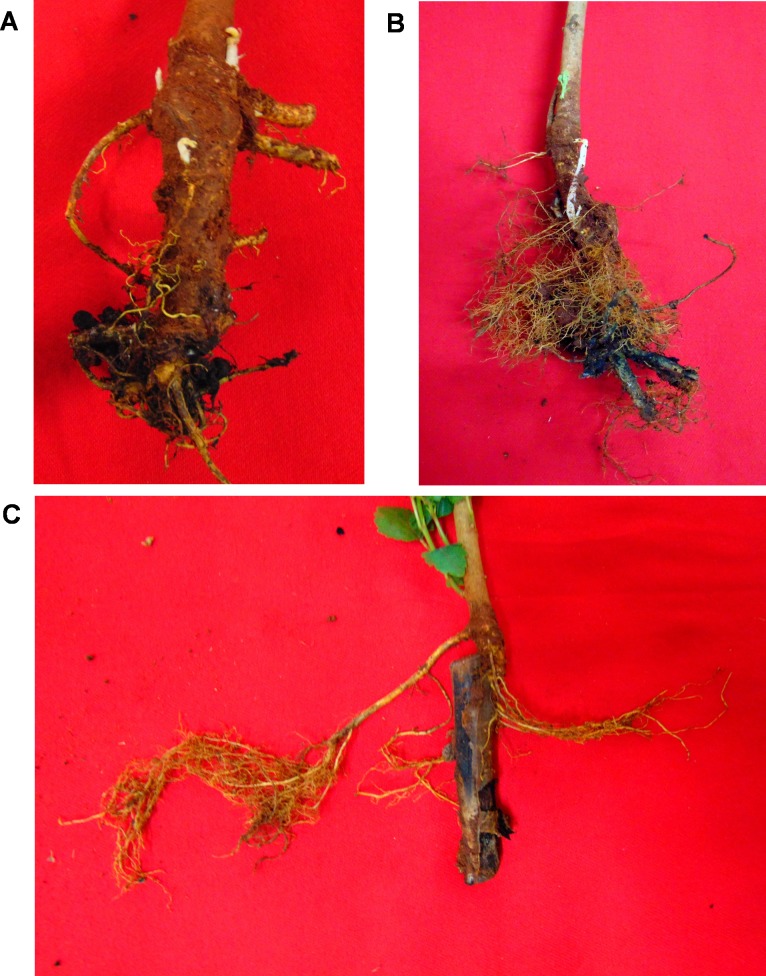
Adventitious shoot (A, B) and root (B, C) regeneration in mulberry infected with *M*. *phaseolina*.

Survival of cuttings and number of roots per sapling were found to be associated with root rot resistance, as these traits had a highly significant negative correlation with leaf wilting, root rot and plant mortality percentages (Fig 2). The survival of cuttings is a measure of the rooting ability of the genotype. Formation of adventitious roots and shoots from the uninfected portions was observed in some resistant and moderately resistant accessions (Fig 9). Du *et al*. [84] reported that many defence-related genes were activated during root formation in mulberry. An increase in sugar transport towards the rooting zone has also been observed during adventitious rooting [85, 86] and may contribute to disease resistance [87]. In sorghum, it was observed that a loss of up to 50% of the roots due to infection had little impact on the leaf water potential [88]. Higher numbers of roots and root regeneration allow plants to sustain water and nutrient mining capability even though parts of the root system are deteriorated due to infection, thereby allowing them to survive. Because the infected plants have to reallocate a substantial amount of photosynthate and stored carbohydrates for regeneration of roots and defence, there will be a loss of vigour. Drought stress and unavailability of nutrition aggravate the problem by decreasing photosynthetic efficiency and render the plants quasi-defenceless. Therefore, mitigation of these stresses by enhancing soil fertility and providing sufficient irrigation becomes crucial in saving the plantation. Nutrient availability is known to prevent pathogenesis [89] and increases the diversity of beneficial soil microflora, which contributes to the control of the pathogen [90]. Furthermore, the survival of cuttings and the number of roots per sapling must be considered as component traits for root rot resistance, and selection of hybrids with higher survival of cuttings and number of roots per sapling must be emphasized in root rot resistance breeding programmes.

### Markers for charcoal rot resistance and associated traits

The charcoal rot resistance trait has a near-normal distribution in the mapping panel, as judged by the distribution of leaf wilting, root rot, healthy root and plant mortality percentages (Table 2). Not much variation in the root traits was present in the mapping panel, as can be inferred from the variance values. This is expected because the mapping panel does not include the poor rooters from the PDG. For association analysis, alleles of all frequencies were retained. Of late, there has been a growing realization that by excluding low frequency alleles, many rare variants are missed out [91, 92]. Tabangin *et al*. [93] found that leaving out such alleles due to concerns about inflating the FDR may not be appropriate, as even alleles with lower frequencies showed type I error rates close to nominal levels. With appropriate measures for controlling FDR, the inclusion of minor alleles can result in the discovery of rare genetic variants underlying complex traits [94]. The Q model was not very effective in controlling FDR as expected because the mapping panel is only weekly structured and most of the accessions are admixtures. K and Q+K models performed adequately in controlling FDR (Table 4, Fig 8). Five markers significantly associated with charcoal rot resistance and four markers for survival of cuttings have been identified (Table 5). No significant MTAs for the number of roots per sapling could be found, perhaps due to a lack of sufficient variability for the trait in the mapping panel. However, Mishra [95] identified RAPD and ISSR markers linked to this trait in the F_1_ mapping population derived from Dudhia White × UP-105 by single marker analysis. Sequencing all these markers and chromosome walking will be useful for elucidating the molecular mechanism of root rot resistance. Conversion of these markers into SCARs or CAPSs will help in efficient and rapid genotyping of germplasm or introgression lines for MAS.

Although mixed-model association mapping approaches are adept at controlling false positives, stringent control for population stratification also results in false negatives [96]. Segregating F_1_ progeny from crosses between resistant and susceptible genotypes can be used to further validate the markers identified in the present study by linkage mapping and to look for additional QTLs. Once validated, breeding resistance to charcoal rot can be expedited by MAS. Based on the disease response, sex expression and genetic dissimilarity, the following parental combinations will be best suited for generation of mapping populations: (1) *M*. *multicaulis* (ME-0006, resistant, ♀) × Thailand Male (ME-0033, highly susceptible, ♂); (2) *M*. *multicaulis* (ME-0168, resistant, ♀) × Thailand Male (ME-0033, highly susceptible, ♂); and (3) Punjab Local (MI-0026, highly susceptible, ♀) × *M*. *cathayana* (Hybrid) (ME-0254, resistant, ♂).

### Future perspectives

It would be oversimplification if we were to assume root rot to be only a host × pathogen interaction. Studies in various crops have clearly demonstrated that the disease outcome is greatly influenced by genotype (host/pathogen) × environment interactions [80, 83, 88, 97, 98]. Previous results have indicated that soil water deficit has a major role in predisposing mulberry to charcoal rot [11], and soil moisture levels below 30% increase disease severity [9]. Drought stress greatly affects mulberry, and leaf yield is reduced to one-third in comparison to the same cultivar gown under optimal irrigation [28]. It has been reported that drought stress deferentially regulates 1051 genes in mulberry [99]. WRKY transcription factors, which play key roles in plant defence signalling and disease resistance [100, 101], were differentially regulated in response to drought stress [99, 102]. The role of these transcription factors in resistance to charcoal rot remains to be investigated.

Harvesting leaves or shoots for silkworm rearing and complete pruning of plants once every 70 days are an integral part of the mulberry cropping system [37]. This means that for 1–2 weeks in each cropping cycle, the plants completely depend on stored nutrients for their survival and regeneration. The effects of repeated pruning on mulberry are not well understood. However, the plants are particularly vulnerable to root rot-causing pathogens due to photosynthetic stress–translocation balance [103]. If environmental stressors act in tandem with pruning stress, it can be highly detrimental to mulberry plantations. Further investigations must be undertaken to understand the pruning stress associated physiological, biochemical and gene expression changes. The effects of moisture deficit, high temperatures, nutritional imbalances and soilborne pathogens in combination with pruning stress also need to be investigated for developing effective strategies to manage the disease.

The wide distribution of root rot-causing pathogens in the South Indian sericulture belt and their genetic diversity [4] are an important source of concern because a dynamic pathogen population is in a perpetual ‘arms race’ and can easily evolve to overcome host disease resistance [104]. A continuous programme for scouting the emergences of new virulent isolates and systematic monitoring of various pathogen populations must be initiated. This will be vital for disease forecasting, taking up adequate control measures and sustaining efforts in genetic improvement of mulberry for root rot resistance.

Though quantitative disease resistance for *Fusarium* spp. and *B*. *theobromae* infection has been reported in mulberry [6, 105, 106], large-scale screening of germplasm for disease response to these pathogens has not been performed. The results obtained in the present study (S5 Table) indicate the availability of genetic resistance to all fungal root rot pathogens in *M*. *multicaulis* (ME-168) and *M*. *cathayana* (Hybrid). Further studies must be initiated to assess the entries in the mapping panel for their reaction to *F*. *solani*, *F*. *oxysporum* and *B*. *theobromae* for identification of accessions with resistance to multiple fungal root rot pathogens. Mining QTLs for resistance to other root rot pathogens or QTLs with pleiotropic effects against multiple pathogens will be useful for breeding durable broad-spectrum resistance by gene pyramiding.

With the advent of next-generation sequencing technologies, comparative transcriptomic studies have been used to understand the molecular mechanisms that regulate abiotic and biotic stress tolerance in mulberry [99, 107, 108]. Similar efforts towards understanding resistance to root rot pathogens will be more comprehensive and will help in the development of functional markers for root rot resistance.

The PDG was established at CSRTI, Mysuru, in 2015 as a part of this study, making the genetic resources available for extensive phenotyping of agronomically important traits. The genotypic information generated on the mapping panel in the present study can be further enriched with additional markers and used to identify QTLs associated with the traits of interest. Utilization of these resources will be efficacious in genetic diversification and improvement of mulberry cultivars.

## Supporting information

S1 Table Estimate of the cost and returns from mulberry sericulture. (DOCX)

S2 Table List of oligonucleotides used in AFLP Genotyping. (XLS)

S3 Table SSR primers screened for marker amplification. (XLS)

S4 Table Details of the mulberry germplasm screened for charcoal root rot resistance. (XLS)

S5 Table Disease responses of the ‘tails’. (DOCX)
