# Supplementary material for: Association mapping of quantitative resistance to charcoal root rot in mulberry germplasm
Source: PLoS One. 2018 Jul 6;13(7):e0200099. doi: 10.1371/journal.pone.0200099 (PMC6034859; doi:10.1371/journal.pone.0200099)
Supplement: S1 Table — (DOCX) [file pone.0200099.s001.docx]

**S1 Table. Estimate of the cost and returns from mulberry sericulture.**

(Data provided by the Sericulture Extension, Economics and Management Division of Central Sericultural Research and Training Institute, Mysuru for the fiscal year 2016–17)

|  | **Particulars** | **Value** |
| --- | --- | --- |
|  | Number of crops per annum | 5 |
|  | Number of DFLs per crop per acre mulberry plantation | 250 |
| **A.** | **Variable costs** |  |
| 1. | Leaf production cost per acre mulberry plantation | ₹ 92,235 |
| 2. | DFLs @ ₹ 5 per DFL | ₹ 6,250 |
| 3. | Disinfectants | ₹ 4,000 |
| 4. | Labour @ 24 man days per 100 DFLs | ₹ 75,000 |
| 5. | Transportation and marketing | ₹ 3,550 |
| 6. | Other expenditures | ₹ 1,000 |
| 7. | Interest on working capital | ₹ 4,369 |
|  | **Total variable costs** | **₹ 1,86,404** |
| **B.** | **Fixed costs** |  |
|  | Depreciation on building and equipments, and interest on fixed costs | ₹ 39,400 |
|  | **Total costs** | **₹ 2,25,804** |
|  | **Cocoon production cost per kg** | **₹ 258** |
| **C.** | **Revenue** |  |
|  | Average cocoon yield per 100 DFLs | 70 kg |
|  | Average cocoon price per kg | ₹ 400 |
|  | Cocoon production per annum per acre mulberry plantation | 875 kg |
|  | Income from cocoons | ₹ 3,50,000 |
|  | Income from by-products | ₹ 17,500 |
|  | **Total revenue** | **₹ 3,67,500** |
|  | **Gross profit** | **₹ 1,41,696** |

DFL, disease free laying.
