# Supplementary material for: Association mapping of quantitative resistance to charcoal root rot in mulberry germplasm
Source: PLoS One. 2018 Jul 6;13(7):e0200099. doi: 10.1371/journal.pone.0200099 (PMC6034859; doi:10.1371/journal.pone.0200099)
Supplement: S5 Table — (DOCX) [file pone.0200099.s005.docx]

**S5 Table. Disease responses of the ‘tails’.**

| **Sl. No.** | **Acc. No.** | **Accession Name** | ***M. phaseolina*** | | ***F. solani*** | | ***F. oxysporum*** | | ***B. theobromae*** | |
| --- | --- | --- | --- | --- | --- | --- | --- | --- | --- | --- |
|  |  |  | **RR** | **DS** | **RR** | **DS** | **RR** | **DS** | **RR** | **DS** |
| 1. | ME-0157 | SRDC-3 | 100 | 5 | 16.31 | 1 | 17.54 | 1 | 42.09 | 2 |
| 2. | ME-0168 | *M. multicaulis* (ME-168) | 12.03 | 1 | 18.73 | 1 | 10.82 | 1 | 16.51 | 1 |
| 3. | ME-0254 | *M. cathayana* (Hybrid) | 9.85 | 1 | 11.55 | 1 | 18.30 | 1 | 45.72 | 2 |
| 4. | MI-0026 | Punjab Local | 100 | 5 | 19.86 | 1 | 15.52 | 1 | 18.48 | 1 |
| 5. | MI-0082 | Acc. 106 | 19.68 | 1 | 47.21 | 2 | 32.89 | 2 | 54.89 | 3 |
| 6. | MI-0421 | Pillighat | 13.20 | 1 | 44.45 | 2 | 80.52 | 4 | 71.64 | 3 |
| 7. | MI-0470 | K2 × BC (P11) | 100 | 5 | 24.86 | 1 | 25.17 | 1 | 25.17 | 1 |
| 8. | MI-0551 | Kollihills-1 | 19.71 | 1 | 47.82 | 2 | 18.66 | 1 | 54.22 | 3 |
| 9. | MI-0775 | Old Jalna | 100 | 5 | 19.89 | 1 | 23.85 | 1 | 27.13 | 2 |
| 10. | MI-0812 | Creeping CP × V1 (P5) | 100 | 5 | 8.87 | 1 | 17.26 | 1 | 16.30 | 1 |

RR, root rot percentage; DS, disease score.
